# Supplementary material for: A deep learning system for detecting diabetic retinopathy across the disease spectrum
Source: Nat Commun. 2021 May 28;12:3242. doi: 10.1038/s41467-021-23458-5 (PMC8163820; doi:10.1038/s41467-021-23458-5)
Supplement: Supplementary file 1 — Supplementary Information [file 41467_2021_23458_MOESM1_ESM.pdf]

A Deep Learning System for Detecting Diabetic Retinopathy Across the Disease Spectrum

Supplementary Information

Supplementary Methods

Utility evaluation of lesion detection and segmentation by healthcare workers

We conducted an experiment to evaluate the utility of our lesion-aware sub-network by measuring its effect on the diagnostic accuracy of trained primary healthcare workers from community health service centers, who were not ophthalmologists. We recruited a group of 20 healthcare workers. The experiment followed a within-subjects design to evaluate the grading performance of primary healthcare workers on a sequence of 500 gradable fundus images (100 images for each diabetic retinopathy (DR) level) randomly chosen from the validation set of the Shanghai Integration Model (SIM) cohort. All the healthcare workers were shown the same set of 500 fundus images, while the order of the images was randomized. For each fundus image shown, the healthcare workers were asked to grade the image into 0 to 4 DR levels (non-DR, mild non-proliferative DR, moderate non-proliferative DR, severe non-proliferative DR, and proliferative DR). After a healthcare worker made a response, the fundus lesion segmentation generated by the DeepDR system was shown overlaid on the fundus image, and he/she was asked the same question on the DR levels again. Ethics approval was obtained from the Ethics Committee of Shanghai Sixth People's Hospital, informed consent was obtained from graders, and graders were deidentified during analyses.

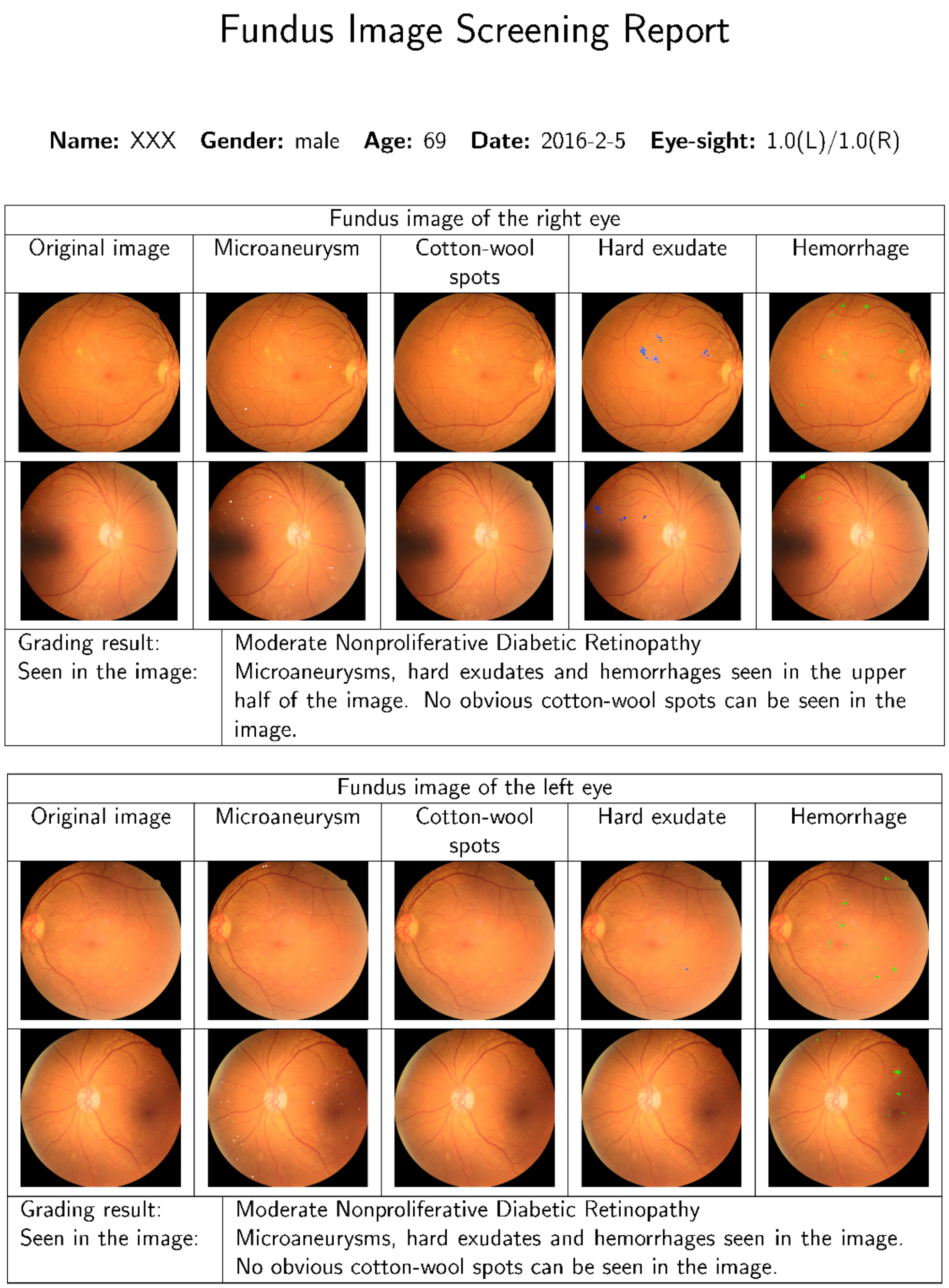

Supplementary Fig. 1. Clinical report automatically generated by the DeepDR system. The report contains visual hints highlight the retinal lesions, description of the presence of the lesions, and diabetic retinopathy grading results.

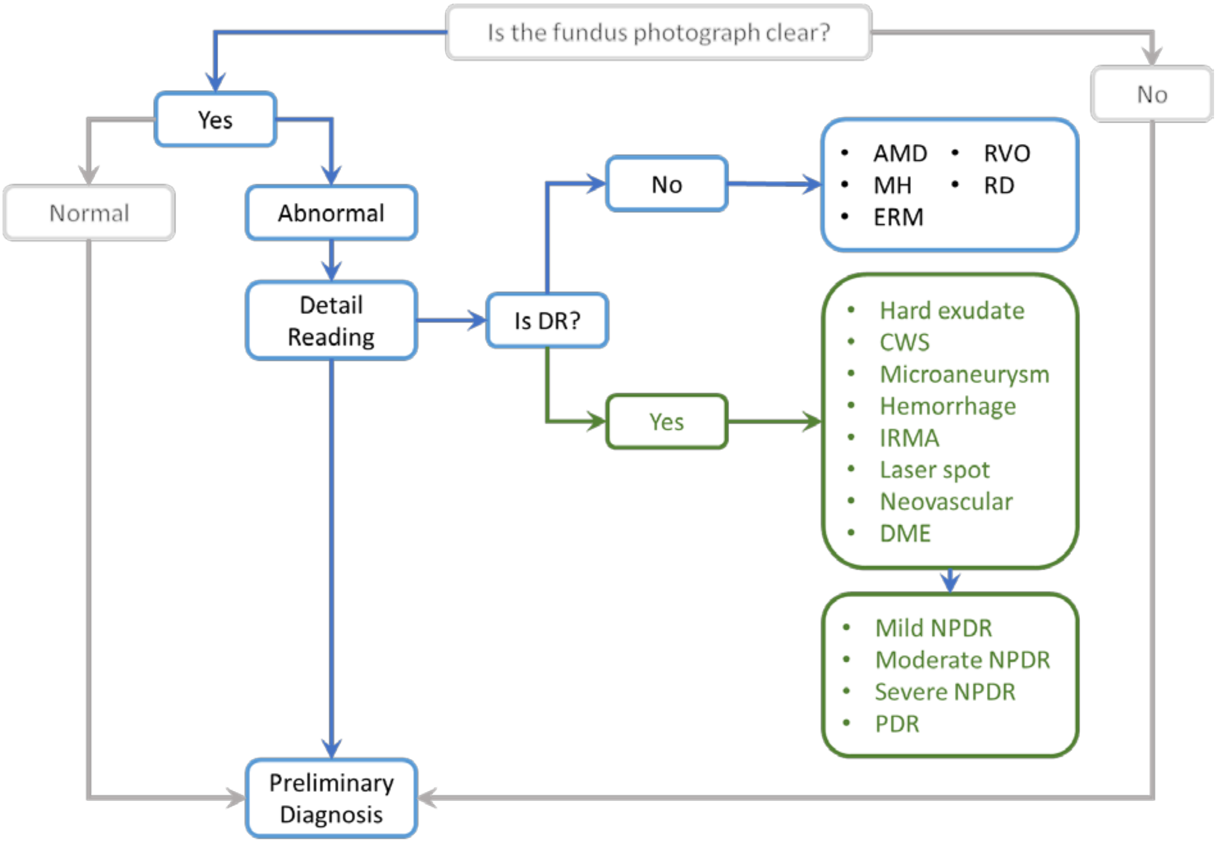

Supplementary Fig. 2. Flow chart of fundus image reading by ophthalmologists. AMD, age-related macular degeneration; CWS, cotton-wool spot; DME, diabetic macular edema; DR, diabetic retinopathy; ERM, epiretinal membrane; IRMA, intraretinal microvascular abnormalities; MH, macular hole; NPDR, non-proliferative diabetic retinopathy; PDR, proliferative diabetic retinopathy; RD, retinal detachment; RVO, retinal vein occlusion.

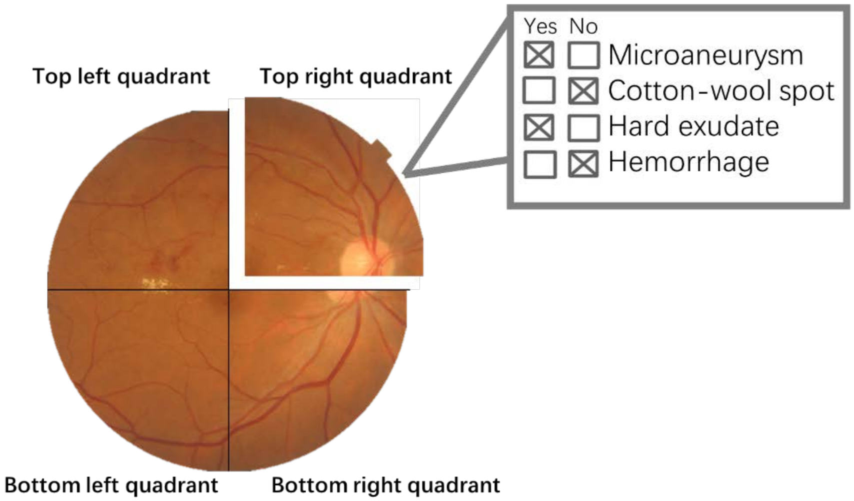

Supplementary Fig. 3. Illustration of lesion detection task. Lesion detection module was a binary classifier that predicted whether any kind of lesions exists in a quadrant (1/4 of the image) of the retinal image.

Supplementary Tables

Supplementary Table 1. Summary of the characteristics of the subjects in the local and external cohorts

	Datasets				
	SIM	CNDCS	P*	NDSP	P*
Year	2014-2017	2018		2018	
Demographics					
No. of subjects	173,346	23,186		6,987	
Age, years	66.22±7.76	62.48±4.23	<0.001	69.11±2.65	<0.001
Male, %	44.60%	42.86%	0.084	44.56%	0.549
BMI, kg/m^2	25.09±3.31	25.14±3.01	0.220	25.34±2.98	0.107
Duration of diabetes, years	7.5 (4.1-12.2)	6.4 (6.3-6.5)	<0.001	5.6 (2.9-10.2)	<0.001
HbA1c, %	7.12±1.26	7.25±1.48	0.003	6.38±1.15	<0.001
Retinal imaging					
Protocol	Two-field 45° images no pupillary dilation	Two-field 45° images no pupillary dilation		Two-field 45° images no pupillary dilation	
Camera	Canon CR-1 Mark II/CR-2; Topcon TRC-NW 200; ZEISS VISUCAM 200	Canon CR-1 Mark II/CR-2; Topcon TRC-NW 200		Topcon TRC-NW400	
Grader experience	A reading group containing 133 certified ophthalmologists (⩾5 years' experience)	A reading group containing 10 certified ophthalmologists (⩾7 years' experience)		A reading group containing 5 certified ophthalmologists (⩾5 years' experience)	

Data are presented as mean±SD or median (interquartile range). *, vs. SIM. Two-sided p value was calculated using t-test for continuous variables and chi-square test for categorical variables. CNDCS, China National Diabetic Complications Study; NDSP, Nicheng Diabetes Screening Project; SIM, Shanghai Integrated Diabetic Prevention and Care System (Shanghai Integrated Model).

Supplementary Table 2. Performance of the lesion-aware module for lesion detection and segmentation in local validation set.

	AUC (95% CI)	Sensitivity (95% CI)	Specificity (95% CI)	Interception over union (IoU) /F-score for microaneurysm
Microaneurysm	0.901 (0.894-0.906)	0.880 (0.872-0.889)	0.733 (0.720-0.743)	0.815
Cotton-wool spot	0.941 (0.935-0.946)	0.900 (0.879-0.919)	0.831 (0.822-0.839)	0.711
Hard exudate	0.954 (0.949-0.957)	0.905 (0.889-0.915)	0.858 (0.852-0.866)	0.971
Hemorrhage	0.967 (0.965-0.969)	0.932 (0.926-0.941)	0.880 (0.876-0.887)	0.738

Supplementary Table 3. Comparison of lesion segmentation results using hard-parameter sharing and soft-parameter sharing

	Hemorrhage (IoU)	Cotton-wool spot (IoU)	Hard exudate (IoU)	Microaneurysm (F-score)
Hard Parameter sharing	0.732	0.707	0.976	0.817
Soft Parameter sharing	0.716	0.703	0.935	0.794
