# Supplementary material for: A deep learning system for detecting diabetic retinopathy across the disease spectrum
Source: Nat Commun. 2021 May 28;12:3242. doi: 10.1038/s41467-021-23458-5 (PMC8163820; doi:10.1038/s41467-021-23458-5)
Supplement: Supplementary file 3 — Reporting Summary [file 41467_2021_23458_MOESM3_ESM.pdf]

nature research

Corresponding author(s): Weiping Jia, Bin Sheng, Haidong Zhou

Last updated by author(s): Feb 22, 2021

Reporting Summary

Nature Research wishes to improve the reproducibility of the work that we publish. This form provides structure for consistency and transparency in reporting. For further information on Nature Research policies, see our Editorial Policies and the Editorial Policy Checklist.

Statistics

For all statistical analyses, confirm that the following items are present in the figure legend, table legend, main text, or Methods section.

n/a	Confirmed	
☐	☒	The exact sample size (n) for each experimental group/condition, given as a discrete number and unit of measurement
☐	☒	A statement on whether measurements were taken from distinct samples or whether the same sample was measured repeatedly
☐	☒	The statistical test(s) used AND whether they are one- or two-sided<br>*Only common tests should be described solely by name; describe more complex techniques in the Methods section.*
☒	☐	A description of all covariates tested
☒	☐	A description of any assumptions or corrections, such as tests of normality and adjustment for multiple comparisons
☐	☒	A full description of the statistical parameters including central tendency (e.g. means) or other basic estimates (e.g. regression coefficient) AND variation (e.g. standard deviation) or associated estimates of uncertainty (e.g. confidence intervals)
☐	☒	For null hypothesis testing, the test statistic (e.g. F, t, r) with confidence intervals, effect sizes, degrees of freedom and P value noted<br>*Give P values as exact values whenever suitable.*
☒	☐	For Bayesian analysis, information on the choice of priors and Markov chain Monte Carlo settings
☒	☐	For hierarchical and complex designs, identification of the appropriate level for tests and full reporting of outcomes
☒	☐	Estimates of effect sizes (e.g. Cohen's d, Pearson's r), indicating how they were calculated

Our web collection on statistics for biologists contains articles on many of the points above.

Software and code

Policy information about availability of computer code

Data collection	NO software was used for data collection.
Data analysis	The code used to train and validate the deep learning system is available on github (https://github.com/drpredict/drpredict).Python version 3.7.1 (Python Software Foundation, Delaware, United States) was used for all statistical analyses in this study. The following third-party python packages were used: OpenCV version 2.4.3 (Intel Corporation, California, United States) was used for image loading and decoding image. Pytorch version 1.0.1 (Facebook, Massachusetts, United States) was used for convolutional neural network computing. Scikit-learn version 0.20.0 (David Cournapeau, California, United States) was used for calculating AUC. Pandas version 0.23.4 (Wes McKinney, Connecticut, United States) was used for loading ground truth and metadata. NumPy version 1.15.4 (Travis Oliphant, Texas, United States) was used for calculating IoU and F-score.

For manuscripts utilizing custom algorithms or software that are central to the research but not yet described in published literature, software must be made available to editors and reviewers. We strongly encourage code deposition in a community repository (e.g. GitHub). See the Nature Research guidelines for submitting code & software for further information.

Data

Policy information about availability of data

All manuscripts must include a data availability statement. This statement should provide the following information, where applicable:

- Accession codes, unique identifiers, or web links for publicly available datasets
- A list of figures that have associated raw data
- A description of any restrictions on data availability

All requests for raw and analyzed data and related materials from the SIM, NDSP, and CNDCS cohorts, excluding programming code, will be reviewed by the Ministry of Science and Technology of China in accordance with the Regulations of the People’s Republic of China on Administration of Human Genetic Resources (State

Council No.717). Any data and materials that can be shared will be released via a Material Transfer Agreement. The EyePACS dataset is publicly available at https://www.kaggle.com/c/diabetic-retinopathy-detection/data.

Field-specific reporting

Please select the one below that is the best fit for your research. If you are not sure, read the appropriate sections before making your selection.

- [x] Life sciences
- [ ] Behavioural & social sciences
- [ ] Ecological, evolutionary & environmental sciences

For a reference copy of the document with all sections, see nature.com/documents/nr-reporting-summary-flat.pdf

Life sciences study design

All studies must disclose on these points even when the disclosure is negative.

Sample size	No sample-size calculation was performed. Sample size was determined by the number of subjects enrolled in the development and validation cohorts. In the development cohort, 173,346 subjects in the Shanghai Integration Model (SIM) cohort who have received fundus examination and have fundus images were enroll in the study. In the validation cohort, 6,987 subjects in the Nicheng Diabetes Screening Project (NDSP) and 23,186 subjects in the China National Diabetic Complications Study (CNDCS) who had received fundus examination and have fundus images before 31 Dec 2018 were enrolled in the study.
Data exclusions	No data were excluded from analysis.
Replication	The DeepDR system was validated in three independent external validation datasets.
Randomization	The primary training dataset (referred to as the local dataset in this study) was divided into development and validation datasets. We obtained a total of 666,383 fundus images in the local dataset from 173,346 patients. Among these, 121,342 subjects (70%) were randomly selected as the training set, and the remaining 52,004 subjects (30%) were served as the local validation set.
Blinding	All ophthalmologists who labeled the fundus images were blinded to the group group allocation and analysis. The investigators were not blinded to group allocation and analysis, because blinding was not relevant to our study due to the inherently blinded nature of test procedures using deep learning algorithm.

Reporting for specific materials, systems and methods

We require information from authors about some types of materials, experimental systems and methods used in many studies. Here, indicate whether each material, system or method listed is relevant to your study. If you are not sure if a list item applies to your research, read the appropriate section before selecting a response.

Materials & experimental systems

n/a	Involved in the study	
x		Antibodies
x		Eukaryotic cell lines
x		Palaeontology and archaeology
x		Animals and other organisms
	x	Human research participants
	x	Clinical data
x		Dual use research of concern

Methods

n/a	Involved in the study	
x		ChIP-seq
x		Flow cytometry
x		MRI-based neuroimaging

Human research participants

Policy information about studies involving human research participants

Population characteristics	SIM cohort consisted of 173,346 diabetic patients with Male percentage 44.6%, average age 66.22±7.76 years, BMI 25.09 ±3.31 kg/m2 and duration of diabetes 7.5 (4.1-12.2) years. CNDCS cohort consisted of 23,186 diabetic patients with Male percentage 42.86%, average age 62.48±4.23 years, BMI 25.14±3.01 kg/m2 and duration of diabetes 6.4 (6.3-6.5) years. NDSP cohort consisted of 6,987 elder subjects with the prevalence of diabetes 31.7%, Male percentage 44.56%, average age 69.11 ±2.65 years, BMI 25.34±2.98 kg/m2 and duration of diabetes 5.6 (2.9-10.2) years.
Recruitment	(1) All subjects who have received fundus examination and have fundus images in SIM; (2) All subjects who have received fundus examination and have fundus images in CNDCS before 31 Dec 2018; (3) All subjects who have received fundus examination and have fundus images in NDSP before 31 Dec 2018. There was no potential self-selection bias to impact results.
Ethics oversight	The study protocol was approved by Ethics Committee of Shanghai Sixth People's Hospital.

Note that full information on the approval of the study protocol must also be provided in the manuscript.

Clinical data

Policy information about clinical studies

All manuscripts should comply with the ICMJEguidelines for publication of clinical research and a completedCONSORT checklist must be included with all submissions.

Clinical trial registration	The study was registered on Chinese Clinical Trial Registry (http://www.chictr.org.cn/). Registration number: ChiCTR2000031184.
Study protocol	The study protocol can be found in the Supplementary Material.
Data collection	Fundus images in the SIM cohort were collected in Shanghai between 2014-2017. Fundus images in the CNDCS cohort were collected in 31 provinces/ autonomous regions/ municipalities in China in 2018. Fundus images in the NDSP were collected in Nicheng Community in 2018.
Outcomes	We compare the diagnostic results made by the deep learning system with the ground truth labeled by ophthalmologists.
